# Three-Dimensional Digital Reconstruction of Ti_2_AlC Ceramic Foams Produced by the Gelcast Method

**DOI:** 10.3390/ma12244085

**Published:** 2019-12-06

**Authors:** Christos S. Stiapis, Eugene D. Skouras, Vasilis N. Burganos

**Affiliations:** 1 Institute of Chemical Engineering Sciences (ICE-HT), Foundation for Research and Technology, Hellas (FORTH), Stadiou, Platani, GR-26504 Patras, Greece; christosstiapis@iceht.forth.gr (C.S.S.); Eugene.Skouras@iceht.forth.gr (E.D.S.); 2 Department of Chemical Engineering, University of Patras, GR-26504 Patras, Greece; 3 Department of Mechanical Engineering, University of the Peloponnese, GR-26334 Patras, Greece

**Keywords:** computational materials engineering, fluid mechanics, advanced geometrical characterization and analysis of porous materials, foams, gelcasting, digital reconstruction

## Abstract

A digital reconstruction technique is presented that generates three-dimensional (3D) digital representations of ceramic foams created by the foam-gelcasting technique. The reconstruction process uses information that is directly extracted from Scanning Electron Microscopy (SEM) images and offers a 3D representation of the physical sample accounting for the typically large pore cavities and interconnecting windows that are formed during the preparation process. Contrary to typical tessellation-based foam treatments, a spherical representation of the pores and the pore windows of the foams is assumed and a novel hybrid algorithm that combines a variation of Lubachevsky-type and Random Close Packing of Hard Spheres (RCPHS) algorithms has been developed to obtain near-optimum solutions to the packing problem of the spheres that represent the pores. Numerical simulations are performed directly on the 3D reconstructed foams to determine their gas permeability. The model predictions are compared with experimental gas permeability data that were obtained for the physical samples. The pore wall thickness can be treated as the single fitting parameter in the entire reconstruction process, although it is shown that images of sufficient resolution could eliminate the need even for that. The foams that are produced by this method yield quantitatively similar pressure drops with experiments for various superficial velocity values, with a very small deviation in the range of 1.7–2.8%. The proposed methodology could be utilized for the prediction of the permeability and transport properties of complex foamy porous structures, similar to the gelcast-type of foams, from a single SEM image of the foam sample without resorting to serial tomography or other structural information, thus saving considerable time and effort from experimental work.

## 1. Introduction

Layered materials are widely available in nature and have been shown to exhibit new and technologically attractive properties when they undergo excessive thinning, delamination or exfoliation, in comparison to their bulk counterparts. As a result, a wide variety of ceramic materials comprised of complex layered structures has appeared, such as MAX phases, characterized by the M_n+1_AX_n_ (n = 1–3) chemical composition formula, where M is an early transition metal, A is an A-group element of the periodic table and X is either C or N [1]. Ti_2_AlC is a member of the MAX-phase group of layered ternary carbides and nitrides. The chemical bonding in Ti_2_AlC is anisotropic in nature and can be described as metal-covalent-ionic because of the distribution of the charge density. Moreover, the chains of directional covalent bonding between titanium and carbon (Ti-C-Ti) and layers of the closed packed Al atoms define the unique properties of Ti_2_AlC that span the group of metals, as well as the group of ceramics [2,3]. Among others, Ti_2_AlC possesses good electrical and thermal conductivity [1], increased resistance to thermal shocks [4] and ease of machining [5], while maintaining its strength at high temperatures [1]. Because of its ceramic nature, it has a very high melting point, a high modulus and a low thermal expansion coefficient [6]. It should be noted that Ti_2_AlC is one of the lightest and most oxidation-resistant [7] carbides reported to date. As a result, porous Ti_2_AlC is considered a promising material for high-performance applications. It can act as an electrode material on rough chemical environments or be utilized as a solar volumetric collector. Moreover, highly porous Ti_2_AlC foams can be used as molds for the production of metal-ceramic interpenetrating composite materials that are characterized by their increased damping properties.

Lately, there has been an increased interest in generating porous Ti_2_AlC foams aiming to produce eventually porous materials with the desired mechanical and transport resistance. The research on porous Ti_2_AlC is considered limited; however, extended studies on those foams may reveal ways to improve their functional properties [8]. Moreover, the permeability of porous Ti_2_AlC at room or higher temperature is a critical feature in various applications, such as catalyst devices and filters [9]. In filters, for example, the performance of gelcast ceramic foams in removing solid particulates from gas flow is highly dependent on the pore window diameter of the foam [10]. In this paper, the focus was placed on Ti_2_AlC produced by the gelcasting method, which is considered a suitable method for obtaining porous materials with a set shape [11]. Gelcasting is a foam production method that is based on the traditional method of forming ceramic materials from casting slips combined with a polymerization reaction. The basic concept of this method involves the production of a ceramic powder suspension with the addition of a substance subject to gelation with the help of a suitable initiator [12]. The mass fraction of the suspension components, as well as the time of the suspension and homogenization process, are suitably selected in order to provide a global time frame that is sufficient to allow suspension to cast into the mold before the gelation process takes place and, at the same time, ensuring that the gelation process does not occur too late [13]. As a last step, the foam samples are placed in an alumina boat on a bed of Al-containing powder [8,14].

Wehinger et al. [15] suggested a methodology for the reconstruction of open cell foams utilizing a Voronoi tessellation, using the pore diameter as the characteristic length. Although they managed to approach experimental data to a great extent using this method, the description that was adopted was limited regarding the shape of the foam ligament/branches. In that approach the branches were assumed to have a constant circular or trapezoidal shape along the strut length. According to observations by Hutter et al. [16], the branch thickness, as well as the branch shape, reportedly affect the mass transport properties of the foam to a large extent. Bracconi et al. [17] and Stiapis et al. [18] attempted to remediate those shortcomings by introducing different branch variation algorithms that shape the strut thickness along the length of branches, greatly improving the description of the solid domain and showing notable agreement with the experimental data. However, these branch variation methods require a predefined arrangement either of points or of non-overlapping spheres, parameters that greatly affect the outcome of the tessellation process. The cells that are created with these methods cannot be defined prior to the tessellation process. The reason is that the cell size distribution generated from the tessellation process is bound to the initial configuration of points or spheres and, thus, rendering the resulting cell size distribution arbitrary to a large extent. Moreover, the cell curvature of the foam pores in a Laguerre tessellation is represented by polyhedral cells that can only approximate geometrical curvatures up to a certain degree. Those shortcomings render the application of Laguerre Tessellations favorable for the reconstruction of foams with specific geometrical structures. To be yet more specific, Laguerre Tessellations are suitable for structures where each cell is interconnected with the others via a single pore window and the cells are preferably not spherical.

In the present work, an algorithm has been developed to create an arrangement of spherically shaped pores that lends itself to the digital reconstruction of the pore structure of Ti_2_AlC foams produced by the gelcasting method with sufficient accuracy. The numerical methods and algorithms that are used in the literature for the generation of dense packings of spheres are classified into two categories—a) the static construction methods and b) the dynamic construction methods. In the static case, the spheres are spatially allocated following a usually sequential deposition process and are considered immobilized after their positioning. In contrast, the dynamic approach uses the initial position of the spheres as an input parameter and assumes short- or long-range interactions between spheres, generating displacements of the spheres and spatial rearrangement of the whole packing, ultimately converging to a packing with the desired properties.

Overall, the static algorithms are very popular due to their direct nature and simplicity. The large value of the foam porosity necessitates the generation of spherical pore packings with a large number density. This can be achieved through several algorithms that are used to generate dense packings of hollow spheres. The majority of those models are based on a simple yet efficient conception, namely that the spheres are placed sequentially in the working domain under the effect of a usually unidirectional force-field and, then, remain fixed in space [19,20]. The static methods, however, can generate relatively homogeneous packings. The packing fractions that are obtained are smaller than the maximum possible fractions for disordered sphere packings [20]. Contrary to the static methods, the dynamic methods are more flexible, albeit more intricate and perplexing. Their complexity is attributed to the fact that the final position of the particles is dependent on the process, which can be either collective or individual. Moreover, the resulting packing fraction strongly depends on the evolution process of the sphere positions.

The dynamic simulation processes are further divided into two major subcategories, namely event-driven simulations and molecular dynamics simulations. In the event-driven simulations, the components of the system under analysis (disks in two dimensions or spheres in three dimensions) evolve independently, unless an event takes place [21]. The sphere evolution is based on the integration of Newton’s equation of motion that is used to predict the trajectory of the spheres [22]. Thus, the temporal displacement of moving spheres is directly proportional to their individual velocity. Collision events interrupt this evolution process and the lineal motion of spheres [22]. These events may correspond to an elastic or inelastic collision between two spheres or to the collision of a sphere with the bounding box walls. The collisions introduce an abrupt interchange on the momentum of the spheres. The duration of body contact during collision is usually assumed to be zero. Using this algorithm, the whole system of spheres is updated after each event [22].

The reconstruction process that is developed here yields three-dimensional, digitized representations of different Ti_2_AlC foams that have different pore size distribution and different porosity values. The final goal of this work is the ability to create 3-dimensional representative volumes of the ceramic foams from minimum input data and to derive useful information regarding their internal structure. Effective transport properties can then be extracted accurately using computational treatment only, thus avoiding exhaustive experimentation. The distinctive advantage of this reconstruction algorithm over other algorithms found in the literature [17,18] is that this algorithm can digitally reconstruct foam structures where each pore cell may be interconnected with others via multiple pore windows and other pore cells. Moreover, this algorithm can be used in order to predict the 3D porosity as a function of the wall thickness of the spherical caps (a quantity that can be easily extracted from Scanning Electron Microscopy (SEM) images). This work aims to speed up the characterization procedure for ceramic foams and provide guidance to the tailoring of their structural properties. A modified event-driven algorithm is proposed, which involves an overlap minimization process that produces an optimized hollow sphere packing. The gas permeability of the reconstructed foams is obtained via numerical methods and is found to compare very satisfactorily with experimental values.

## 2. Materials and Methods

In this work, a methodology for the 3-dimensional reconstruction of dry foams is developed, followed by the numerical extraction of their effective flow and transport properties. The reconstruction approach is based on a packing of non-overlapping or partially overlapping spheres, whose radii are sampled from the size distribution that is extracted by pore-space analysis on a single SEM image.

Depending on the application, some disk-packing or sphere-packing generation algorithms are found to perform better than others in some aspects of the simulation procedure yet fall behind in some other aspects. Thus, it is desirable to combine algorithms in a way that exploits their strengths and manages to sidestep their weaknesses, formulating a new, hybrid, “composite” algorithm. The hybrid algorithm that is developed here consists of two well-established algorithms for the generation of a hollow sphere packing. The first constituent algorithm is the algorithm proposed by Lubachevsky who worked first on billiard simulations [23] and then on random disk packing problems [24]. The second constituent algorithm is the Random Close Packing of Hard Spheres algorithm (RCPHS) developed by Wu et al. [25], which is a rearrangement algorithm with an optimization subroutine.

The Lubachevsky algorithm uses the concept of following the motion of a number of spheres in a closed domain, similar to a billiard simulation. However, the diameter of the spheres grows gradually in time. Since the volume of the domain is finite, there will be a time when the spheres cannot grow any further, yielding a jammed pack. This algorithm has one parameter representing the growth rate of the spheres compared to the mean velocity of the spheres. In practice, low values of the growth rate parameter are associated with sphere packing that tend to approach the ordered state; however, for larger values of the growth rate a more chaotic dispersion is attained. The Lubachevsky algorithm also has the appealing characteristic of producing dense jammed packaging for monodispersed sphere systems; however, this claim cannot be extended to polydispersed sphere systems [26]. Moreover, due to the fact that this algorithm has been strictly designed for hard spheres, it is not capable of handling jammed packings of soft, partially overlapping spheres without inherent modifications.

The RCPHS algorithm considers an arrangement of spheres with overlap between neighboring spheres. Then, a procedure is applied to relocate each sphere so that overlapping is reduced. When the total sphere overlapping volume cannot be further reduced, all spheres are incrementally shrunk. By consecutive repetition of the relocation and shrinking steps, a non-overlapping or, in any case, a minimized overlapping packing is eventually obtained. However, in the core of this algorithm lies a shrinking step that manipulates the radius of the spheres in order to facilitate the minimization of the overlapping between the spheres. The shrinking step of this algorithm is undesirable in our implementation since the resulting distribution of the sphere radii is not guaranteed to follow the desired one.

### 2.1. Proposed Hybrid Algorithm for Packing Generation

In this work, a hybrid algorithm that combines a variation of the Lubachevsky and the RCPHS algorithms is developed to obtain a near-optimum solution to the sphere packing problem. Because of the fact that the RCPHS algorithm requires an initial arrangement of spheres in order to operate on them and minimize their overlap, a variation of the Lubachevsky-Stilling [23] algorithm is used. At first, the simulation domain is defined paying attention to the conditions that should apply at the boundaries. The spheres can either rebound off the walls of the simulation domain (rigid boundary) or pass through the wall to re-enter through the opposite side (periodic boundary). In the present case, the rigid boundary condition is adopted since it combines robustness and simplicity.

Then, the simulation domain is populated by spheres that are randomly placed in space and have as radii a very small fraction of the finally desired ones. Their velocity components are represented by randomly oriented unit vectors. To simplify the computations, the radii and positions are further normalized by the characteristic domain length. The spheres move with the unit velocity into random directions and are subject to elastic collisions with other spheres or with the domain boundaries. As the sphere sizes gradually increase (exponential growth is assumed) and the domain size is fixed, there will be a moment at which a jammed configuration is obtained. More specifically, starting from a random spatial distribution of very small spheres, they are allowed to transform gradually into non-overlapping or partially overlapping spheres, the radius of which is increased at a constant expansion rate, defined as *b_rate_*, while their positions evolve over time according to Newtonian mechanics. The expansion of the spheres is assumed to occur simultaneously on all spheres after every collision event. Thus, as time progresses, each sphere experiences an increasing number of collisions due to the fact that the available free space is reduced gradually. When a jammed stage is reached and no further repositioning according to the previous rules is possible, the spheres are magnified to their original (desired size). The RCPHS algorithm is subsequently employed to minimize the overlapping of spheres by rearranging their positions.

#### 2.1.1. Sphere Collisions

When the spheres are not colliding, they move in straight paths; no forces act on them and there is no acceleration. The collision time between two spherical particles can be determined as follows. Let us assume two spheres *i* and *j* with radius *R_i_* and *R_j_* and velocity ${\vec{v}}_{i}$ and ${\vec{v}}_{j}$, respectively. If these two spheres are in a collision trajectory, they are going to collide at time $\Delta t_{c}$ and the following equation will be satisfied, Equation (1).

(1) $$\Delta t_{C}^{2}{\left|{\vec{v}}_{ij}\right|}^{2}+2\Delta t_{C}\left({\vec{v}}_{ij}\cdot {\vec{r}}_{ij}\right)+{\left|{\vec{r}}_{ij}\right|}^{2}-\sigma_{ij}^{2}=0$$

*σ_ij_ = R_i_ + R_j_* is called the interaction diameter, ${\vec{r}}_{ij}$ is the relative position vector between spheres *i* and *j* and ${\vec{v}}_{ij}$ is their relative velocity. This quadratic equation in $\Delta t_{c}$, Equation (1), is obtained by squaring the vectorial expression for the binary collision of spheres with constant velocity in time. However, during the computation of the roots of Equation (1), numerical errors accumulate during the calculation, attributed to the finite precision of floating point calculations. These errors, if left untreated, inevitably lead to negative time-steps or complex solutions of the quadratic. They also affect the prediction of the positions of the colliding spheres at the time of impact, which results either in spheres overlapping or in positioning of spheres at a small distance from each other or from the wall, at the time of collision. The sphere overlapping case is extremely problematic [22,27], since the particles have numerically entered the infinite energy hard-core [27]. Errors resulting from $\left|{\vec{r}}_{ij}\right|\;\geq R_{i}+R_{j}$ do not affect the stability of the algorithm. In order to deal with the floating point precision problem, the algorithm that was proposed by Bannerman et al. [18] is implemented. In this algorithm, an overlap function, $F_{HS}$, is used at any time step, defined as the left-hand-side of Equations (1) and shown in Equation (2),

(2) $$F_{HS}=\Delta t_{C}^{2}{\left|{\vec{v}}_{ij}\right|}^{2}+2\Delta t_{C}\left({\vec{v}}_{ij}\cdot {\vec{r}}_{ij}\right)+{\left|{\vec{r}}_{ij}\right|}^{2}-\sigma_{ij}{}^{2},$$

which evaluates the overlapping between two spheres at each time step. This overlap function obtains negative, positive or zero values for a pair of overlapping, non-overlapping or one-point contacting spheres, respectively. Thus, the problem of sphere collisions is transformed into a problem of finding the roots of F*_HS_* = 0. The derivative of the overlap function with respect to the collision time may be utilized in order to predict future overlapping between spheres that are not currently overlapping. By considering the aforementioned properties, overlap in each pair occurs when. This overlap decreases, that is, the spheres move away from each other, when $\partial F_{HS}/\partial \Delta t_{c}>0$. Conversely, the spheres approach each other and the overlap increases when $\partial F_{HS}/\partial \Delta t_{c}<0$. Thus, the search for the earliest occurring collision pair is led by the smallest non-negative $\Delta t_{c}$ that is subject to the overlap function conditions:

(3) $$\partial F_{HS}\left(t+\Delta t_{c}\right)\leq 0$$

(4) $${\frac{\partial F_{HS}}{\partial \Delta t_{c}}|}_{t+\Delta t_{c}}<0.$$

Thus, by following the definition of the overlap function and employing the following algorithm sequentially, for each pair of spheres *i*, *j* the $\Delta t_{c,i,j}$ is calculated, which is the time increment for the mutual collision of the spheres.

(1) If $\;{\vec{v}}_{ij}\cdot {\vec{r}}_{ij}\geq 0$ then $\Delta t_{c,i,j}\to \infty$.
(2) Else if ${\left|{\vec{r}}_{ij}\right|}^{2}-\sigma_{ij}{}^{2}\leq 0$ then $\Delta t_{c,i,j}=0$.
(3) Else if ${\left({\vec{v}}_{ij}\cdot {\vec{r}}_{ij}\right)}^{2}-{\left|{\vec{v}}_{ij}\right|}^{2}\left({\left|{\vec{r}}_{ij}\right|}^{2}-\sigma_{i,j}^{2}\right)\leq 0$ then $\Delta t_{c,i,j}\to \infty$.
(4) If none of the above is satisfied, then Δ*t*_c_ is calculated from
  $$\Delta t_{C,i,j}=\frac{{\left|{\vec{r}}_{ij}\right|}^{2}-\sigma^{2}}{-\left({\vec{v}}_{ij}\cdot {\vec{r}}_{ij}\right)+\sqrt{{\left({\vec{v}}_{ij}\cdot {\vec{r}}_{ij}\right)}^{2}-{\left|{\vec{v}}_{ij}\right|}^{2}\left({\left|{\vec{r}}_{ij}\right|}^{2}-\sigma_{ij}{}^{2}\right)}}.$$

At the end of the execution of this algorithm, a list of the mutual collision times is formed. In addition to the fact that the spheres are placed inside an orthogonal computational domain, the collision times with the domain walls are also calculated, $\Delta t_{c,w}$. The next colliding interval is determined as the minimum positive value of both lists:

(5) $$\Delta t_{c}=min\left(\Delta t_{c,i,j}-\Delta t_{c,w_{i}}\right),\;\Delta t_{c,i,j}>0.$$

The spheres are allowed to move forward in time by a $\Delta t_{c}$ time-interval. The collision time, $t_{c}=t+\Delta t_{c}$, represents the moment of collision between a pair of spheres or a sphere-wall collision. Thus, in the next step the pair collision needs to be resolved, to yield the outcome of the collision and define the starting condition for the next rearrangement stage.

#### 2.1.2. Collision Handling

The sphere-to-sphere collisions, as well as the sphere-to-wall collisions, are very fundamental for this algorithm, as they determine the sequential events of the simulation. The classical elastic collisions type is adopted here. However, this property is not ensured since each sphere is gradually growing and, thus, there is an outward surface velocity relative to the sphere center. A corrective algorithm has been developed in order to treat this peculiarity, described in Section 2.1.5. The following two equations are obtained by the employment of the conservation of momentum and kinetic energy for the two colliding spheres:

(6) $$\Delta {\vec{p}}_{m}=\Delta p_{m}{\hat{n}}_{ij}=m_{i}\left({\vec{v}}_{i}^{*}-{\vec{v}}_{i}\right)=-m_{j}\left({\vec{v}}_{j}^{*}-{\vec{v}}_{j}\right)$$

(7) $$m_{i}\left({\vec{v}}_{i}^{*}-{\vec{v}}_{i}\right)=\Delta p_{m}{\hat{n}}_{ij}\to {\vec{v}}_{i}^{*}={\vec{v}}_{i}-\left(\frac{\Delta p_{m}}{m_{i}}\right){\hat{n}}_{ij},$$

where $\Delta {\vec{p}}_{m}$ is the momentum change of each colliding sphere, ${\vec{v}}_{i}^{*}$ and ${\vec{v}}_{j}^{*}$ are the post-collisional velocities of spheres *i* and *j* and ${\hat{n}}_{ij}$ is the unit vector directed from the center of sphere *i* to the center of the sphere *j*. However, ${\hat{n}}_{ij}\cdot {\hat{n}}_{ij}=1$, thus Equation (7) is reformulated into Equation (8):

(8) $$\Delta {\vec{p}}_{m}=\frac{2m_{i}m_{j}}{m_{i}+m_{j}}\left(\frac{\left({\vec{v}}_{ij}\cdot {\vec{r}}_{ij}\right){\vec{r}}_{ij}}{\sigma_{ij}^{2}}\right).$$

Assuming that all spheres in the simulation domain are identified with the same unity mass although they may have different volume, the expression for the momentum change reads:

(9) $${\vec{v}}_{i}^{*}={\vec{v}}_{i}-\left(\frac{\left({\vec{v}}_{ij}\cdot {\vec{r}}_{ij}\right){\vec{r}}_{ij}}{\sigma_{ij}^{2}}\right).$$

**Figure 1:**
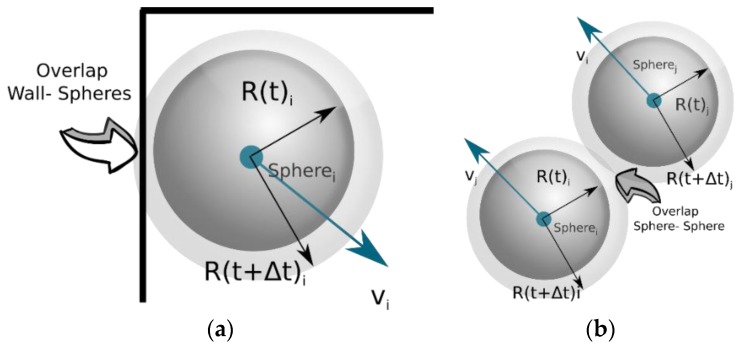
Artificial overlapping introduced by the gradual increase in the sphere radius. (**a**) Walls-to-sphere overlapping; (**b**) Sphere-to-sphere overlapping.

In this way, all spheres are allowed to experience the same domain volume irrespective of their size. At the end of each collision step, all spheres have their radii increased by the *b*_rate_ factor. This will inevitably create overlaps between the spheres, as well as between the spheres and the walls. In this study, *b*_rate_ assumes a value of 1.00005 that is translated to an increase of 0.005% in all sphere radii after each collision event. This value was found to be optimal for the present simulations. Very small values of *b*_rate_ would result in significantly elevated running time without any noticeable change in the structure features. On the other hand, simulations with larger *b*_rate_ values would result in encapsulation of small spheres by larger ones, an unfavorable situation for the algorithm. The reason behind those overlaps lies in the fact that, when the spheres are colliding and their post-collision velocities are calculated, their radius is incrementally increased, creating an artificial overlap, as shown in Figure 1a,b, which is inevitable especially when a jammed state is reached while the spheres resume their actual, desired size.

In order to resolve those overlaps, a local repositioning algorithm is devised to relocate each sphere to a new position and reduce the overlap. In the present work, a modification of the rearrangement algorithm by Wu et al. [25] is proposed. The spheres are moved sequentially in order to reduce the total overlapping volume gradually. Only one sphere may be moved in each attempt, while the rest of the spheres are assumed immobilized. To this end, an optimization routine is employed that searches for a new position for the sphere under consideration within a distance of twice the sphere radius from the original position of the sphere. The new position should minimize its overlapping volume with the surrounding spheres. The overlapping volume between a pair of spheres is a non-linear function of their relative coordinates, Equation (10):

(10) $$V_{i,j}^{o}\left({\vec{r}}_{ij}\right)=\{\begin{array}{cc} \sqrt{\pi }\left(R_{i}+R_{j}-\left|{\vec{r}}_{ij}\right|\right)\frac{\left({\left|{\vec{r}}_{ij}\right|}^{2}+2\left|{\vec{r}}_{ij}\right|R_{j}-3R_{j}^{2}+2\left|{\vec{r}}_{ij}\right|R_{i}+6R_{i}R_{j}-3R_{i}\right)}{12\left|{\vec{r}}_{ij}\right|} & ,if\;\left|{\vec{r}}_{ij}\right|<R_{i}+R_{j}\\ \frac{4\pi R_{j}^{3}}{3} & ,if\;R_{i}>\left|{\vec{r}}_{ij}\right|+R_{j}\\ \frac{4\pi R_{i}^{3}}{3}\;\;\; & ,if\;R_{j}>\left|{\vec{r}}_{ij}\right|+R_{i}\\ 0 & ,if\left|{\vec{r}}_{ij}\right|>R_{i}+R_{j} \end{array}$$

Thus, the total overlapping volume of a sphere with the rest of the N spheres is given from Equation (11):

(11) $$V_{i}^{T}=\sum_{j=0}^{j=N,j\neq i}\left(V_{i,j}^{o}\left({\vec{r}}_{ij}\right)\right).$$

This process aims to the minimization of the total overlapping volume, treated as an objective function, over a set of decision variables under a set of constraints. The minimum-overlap problem can thus be formulated as follows:

(12) $$\underset{{\vec{x}}_{i}}{\min}\;\;\;V_{i}^{T}\left({\vec{x}}_{i}\right),$$

(13) $$\mathrm{subject}\;\mathrm{to}\;R_{i}<x<L_{domain}-R_{i}\;,\;\forall \;x\in \;\vec{x}.$$

This formulation transforms the problem of sphere-to-sphere overlapping to a problem of coordinates. Thus, the query to be addressed is now converted to the following—what coordinates of the center of sphere *i* will minimize its overlap with the rest of the spheres *j*. The optimizer that is used to solve the minimization problem is a Sequential Least-Squares Programming algorithm (SLSQP) [28] that was developed based on the least-squares solver of Lawson and Hanson [29]. The minimization of the overlapping volume concludes either when a predefined threshold fraction of the overall domain volume is reached or when no further overlap reduction is observed. The threshold fraction is set to 10^−6^, as a compromise between speed and accuracy. Smaller threshold values have also been tested but they were found to increase the computational burden substantially without improving the accuracy in practice. The algorithm optimizes successive second-order (quadratic/least-squares) approximations of the objective function via updates, with first-order approximations of the constraints. Moreover, this method was applied to aerodynamic and robotic trajectory optimization by Kraft [28].

#### 2.1.3. Selection of Sphere to Relocate

**Figure 2:**
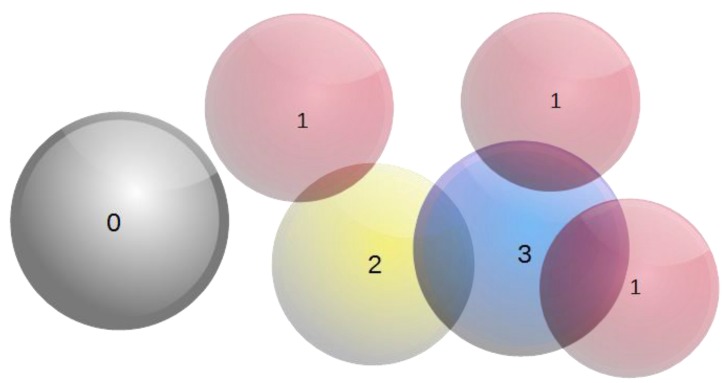
Assigning weights on spheres based on their overlapping state.

The selection of the sphere to be relocated is made with a probability that is proportional to the count of the overlaps with neighboring spheres. Hence, spheres that contribute greatly to the overlapping phenomenon are prioritized in the relocating process. To this end, a weight is assigned to each sphere, indicating the overlap count. Repeating this process for all the spheres in the simulation domain provides a distribution of the overlap count that all spheres exhibit with their neighboring spheres, Figure 2.

#### 2.1.4. Generation of the Micro-Voids

Observation of several SEM images reveals that the TI_2_AlC foams produced with the gelcasting method are comprised, typically, by nearly spherical cells connected by circular windows [30]. In the current approach, the pore windows are simulated as circular cutouts on the spherical cells. Such small circles can be created either by intersecting a plane with the pore cells or with spheres whose centers lie on the surface of the cell. In the present algorithm, the pore windows are generated by creating an arrangement of non-overlapping small spheres on the surface of each pore cell. Alternatively, this can be portrayed as the packing of different caps on the pore cell surface. This approach allows one to translate the problem of pore windows generation into a case of non-overlapping packing of small spheres on a large sphere surface, as described next.

#### 2.1.5. Non-Overlapping Packing of Spheres on a Sphere Surface

The pore windows in each pore cell is sequentially generated in the present algorithm. Initially, the number and size of pore windows on each pore cell surface are extracted from the SEM image and their frequency distributions are constructed. During the reconstruction process, the number and the size of the pore windows are sampled from this distribution for the specific pore cell size at hand. Initially, the sphere arrangement representing the pore windows on the surface of pore cells takes place using a random process. By iterating and minimizing the overlaps of the spheres on the surface of the macrovoids, an overlap-free arrangement of spheres on the surface of the cell is created. This is performed by initially depositing randomly the sphere centers ensuring a uniform initial distribution on the surface of each pore cell. The minimization of the overlapping function is performed using the differential evolution algorithm [31].

However, there is a significant probability that the resulting macro-voids overlap with each other, creating extra spherical caps that would create extra pore windows connecting the macrovoids, a process that must be included in the reconstruction process. In order to generate pore cells that would contain the exact number and size of the pore windows extracted from the SEM, the radius of the spherical cap that relates to the overlapping of the pair of macrovoids is computed. Then, from each overlapping macrovoid the sampled pore windows sizes to be deposited on their surface are modified. The modification process includes the assignment of the radius of the spherical cap that is related to the pair of the overlapped macrovoids to the pore window with the closest radius to it. Moreover, the position of the sphere- representing the pore window connecting two macrovoids is fixed according to Equation (14)

(14) $${\vec{x}}_{Pore\;window}=\frac{R_{\mathrm{mv},2}{\vec{x}}_{\mathrm{mv},1}+R_{\mathrm{mv},1}{\vec{x}}_{\mathrm{mv},2}}{R_{\mathrm{mv},1}+R_{\mathrm{mv},2}},$$

where *R*_mv,1_, *R*_mv,2_ are the radii of the overlapping macrovoids and ${\vec{x}}_{\mathrm{mv},1}$, ${\vec{x}}_{\mathrm{mv},2}$ the positions of the centers of the two overlapping macrovoids.

#### 2.1.6. Thickness of the Macrovoids

**Figure 3:**
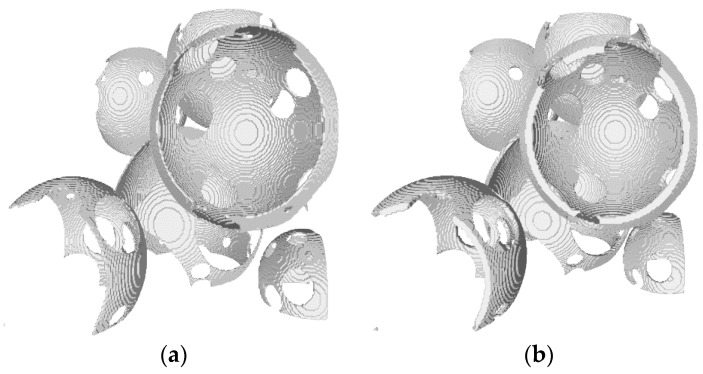
The encapsulation of the sphere with a spherical shell (**a**) with a shell of smaller thickness; (**b**) with a shell of increased thickness.

An arrangement of permeable hollow spheres is obtained after the last step. At the end, the thickness of all hollow spheres is adjusted to achieve the desired foam porosity. Alternatively, the shell thickness can be extracted from the SEM image and used as input to the algorithm. In this case the porosity will be estimated directly from the algorithm bypassing the need for its experimental determination. The capability of the proposed algorithm to offer a reliable estimate of the foam porosity is discussed in Section 3.3. The effect of pore wall thickness on the foam reconstruction is portrayed in Figure 3a,b.

#### 2.1.7. Flowchart of the Algorithm

**Figure 4:**
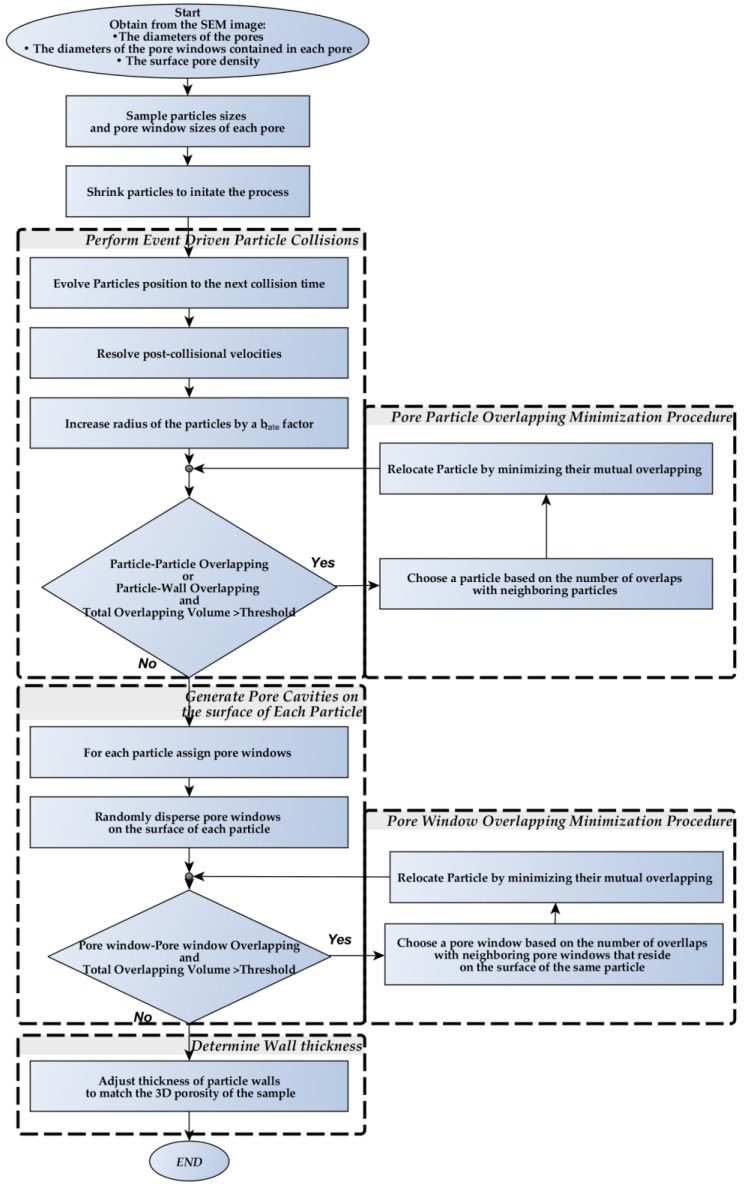
Flowchart for the proposed reconstruction method.

The flowchart of the reconstruction algorithm proposed in this work is presented in Figure 4. The algorithm begins by extracting statistical information from the SEM image of the porous media and its execution provides the user with a 3D digital reconstruction of the porous media under consideration. The algorithm proceeds by performing three main sequential steps, namely (i) the creation of a packing of hollow spheres representing the pores, where the pair overlaps are minimized, (ii) generation of pore windows achieved by the deposition of non-overlapping spheres with traces on the surface of each hollow sphere and (iii) adjustment of the thickness of the pore walls to match the porosity of the real sample.

### 2.2. Permeability and Tortuosity Estimation

#### 2.2.1. Pressure Drop

In recent years, a number of studies have shown the importance of determining the various flow regimes before evaluating the Darcy permeability, as well as the inertia correction coefficient [32]. Depending on the flow regime, namely, Darcy, inertial or turbulent, different macroscopic equations must be used and different flow coefficients must be evaluated. A common practice that is followed in the literature to define the flow regime is the graphical method, which is also adopted here and is briefly described in Section 2.2.5.

#### 2.2.2. Flow Simulation

Since the present work focuses mainly on the resolution of flow inside the porous medium, employment of a detailed description of the flow inside the porous medium at pore-level was chosen. The flow characteristics are discussed in Section 3.1.3 using the characteristic numbers for the specific foam structures. Because of the very low values of the Knudsen number, well below the threshold limit of 0.01, the continuum approach was adopted. In the continuum flow regime the thermodynamic equilibrium near the walls and the no-slip boundary conditions are inherently valid [11]. Since the Reynolds number was on the order of unity, the laminar regime was also adopted for the viscous description and the momentum and mass conservations were described by the Navier-Stokes equations. Gas compressibility effects were also non-critical due to the very low Mach number of the systems. In the work by Akbarnejad et al. [33], the effect of fluid flow on the resulting pressure gradient was studied during permeametry experiments on unsealed ceramic foam filters. They empirically derived Darcy and non-Darcy permeability coefficients based on Forchheimer’s equation. Their simulations were performed in the turbulence flow regime. Flow in the porous domain was described with a Reynolds-Averaged Brinkman–Forchheimer equation without resorting to pore-level description.

OpenFOAM [34,35] was used here to simulate the gas flow in the reconstructed porous domains. OpenFOAM is an open source Computational Fluid Dynamics (CFD) software package that solves complex fluid flows using the Finite Volumes method and handles chemical reactions, turbulence and heat transfer. It uses various CFD algorithms for laminar or turbulent flow modeling. The rhoSimpleFoam [35] has been utilized, which is a steady-state solver used to simulate compressible Reynolds-Averaged Navier-Stokes (RANS) flows employing a pressure-based (predictor-corrector) approach and the Semi-Implicit Method for Pressure Linked Equations (SIMPLE) algorithm [36,37]. This solver offers an evaluation of the mass flow and the temperature variation throughout the working domain. In this context, the conservative, differential form of the 3D compressible Navier-Stokes (N-S) equations is solved. The N-S equations regulate the preservation of the compressible fluid flow mass, momentum and energy and are shown in Equations (15)–(17):

(15) $$\nabla \cdot \left(\bar{U}\rho \right)=0$$

(16) $$\nabla \cdot \left(\vec{U}\left(\rho \vec{U}\right)\right)+\nabla p+\nabla \cdot \bar{\bar{\sigma_{f}}}=0$$

(17) $$\nabla \left(\vec{U}\left(\rho E\right)\right)+\nabla \left(\vec{U}\vec{p}\right)+\nabla \cdot \left(\bar{\bar{\sigma_{f}}}\cdot \vec{U}\right)+\nabla \cdot \vec{J_{t}}=0,$$

where *U* represents any Cartesian component of the local fluid velocity, *p* stands for the pressure of a fluid element, *ρ* is the density and $E=e+{\left|U\right|}^{2}/2$ indicates the total specific energy with *e* the internal specific energy. The closure of the set of partial differential equations is accomplished with three common assumptions regarding the thermodynamic and kinetic nature of the gas, which correspond to the ideal gas assumption, the calorically perfect gas assumption and the Newtonian fluid assumption. The first two expressions are represented by Equations (18) and (19):

(18) $$p=\left(\frac{c_{p}}{c_{v}}-1\right)\rho e$$

(19) $$e=c_{v}T,$$

where *c_p_* and *c_v_* are the specific heat of the gas at constant pressure and constant volume, respectively. The heat flux used in Equation (17) is calculated considering the Fourier law, expressed in Equation (20):

(20) $${\vec{J}}_{t}=-a\nabla T=-c_{p}\frac{\mu }{\mathrm{Pr}}\nabla T$$

where *μ* represents the dynamic viscosity of the gas, α is its thermal conductivity and Pr is the Prandtl number defined for laminar flows. The components of the viscous term tensor are given by

(21) $$\bar{\bar{\sigma_{f}}}=-2\mu \left(\frac{1}{2}\left(\nabla \vec{U}+{\left(\nabla \vec{U}\right)}^{T}\right)-\frac{1}{3}tr\left(\frac{1}{2}\left(\nabla \vec{U}+{\left(\nabla \vec{U}\right)}^{T}\right)\right)\bar{\bar{I}}\right),$$

where $\bar{\bar{I}}$ is the unit tensor.

#### 2.2.3. Transport Properties

In order to solve the energy equation, definition of the fluid properties is necessary, namely, the viscosity, *μ*, the specific heat, *c_p_* and the thermal diffusivity, α. For the sake of comparison with experimental data, the present simulations address the isothermal flow of Argon gas close to the ambient pressure, at T = 279 K in Gelcast93 and Gelcast87 samples and at T = 973 K for the case of the Gelcast84 sample. These properties for the flowing Argon gas are assumed to be constant. Therefore, the following values for the dynamic viscosity, the heat capacity and the Prandtl number are used—*μ*_279 K_ = 2.24 × 10^−5^ (kg m^−1^ s^−1^), *c_p,_*_279 K_ = 520 (J kg^−1^ K^−1^), Pr_279 K_ = 0.66 [38,39], *μ*_973 K_ = 5.44 × 10^−5^ (kg m^−1^ s^−1^) [40] *c_p,_*_973 K_ = 520 (J kg^−1^ K^−1^), Pr_973 K_ = 0.66 [38].

#### 2.2.4. Boundary Conditions

The boundary conditions complementing the transport equations are reported next.

- Inlet—a flat velocity profile is imposed, corresponding to a fixed volumetric flowrate entering the domain equal to the flowrate reported in the experiments. Argon is assumed to enter the porous domain at a constant static temperature of T_0_ = 297 K for Gelcast93 and Gelcast87 and T_0_ = 973 K for Gelcast84.
- Walls—since the pore walls are assumed to be immobile, every velocity component at the walls is set to zero, as in no-slip conditions. The zero gradient boundary condition is specified for the pressure and the walls are assumed to have the same temperature as their environment, thus a constant static temperature is prescribed on them.
- Outlet—since the foam is open to the atmosphere and no recirculation is expected, a Neumann boundary condition is used for the velocity there. A uniform fixed value of 1 atm is assumed for the pressure. The zero gradient boundary condition is imposed on the temperature.
- Domain faces parallel to the flow direction—the normal derivatives of the velocity, pressure and temperature are set to zero.

#### 2.2.5. Graphical Estimation

In the graphical method the reduced pressure gradient is plotted against the superficial velocity and the form of the curve reveals the flow regime of interest. In the Darcy regime, the pressure drop is prescribed by the linear Darcy equation, shown in Equation (22):

(22) $$\frac{\Delta P}{L}=\frac{\mu }{K_{D}}\langle \vec{U}\rangle ,$$

where Δ*P* is the macroscopic pressure drop and *L* is the medium thickness. However, in the strong inertia regime the quadratic Forchheimer equation is known to hold, shown in Equation (23):

(23) $$\frac{\Delta P}{L}=\frac{\mu }{K_{F-D}}\langle \vec{U}\rangle +\frac{\rho }{K_{F-F}}{\langle \vec{U}\rangle }^{2}.$$

It should be noted that the quantity $K_{F-D}$ is usually referred to as Darcian permeability; however, it is not the same as the Darcy permeability, $K_{D}$, used in Equation (22).

#### 2.2.6. Representative Elementary Volume (REV) Analysis

A Representative Elementary Volume (REV) analysis has been conducted for each reconstructed foam, namely for the Gelcast84, Gelcast87 and Gelcast93 foams, by calculating the porosity using gradual increments in the REV volume. Every control-volume was cubic in shape and different control volumes were obtained by increasing the edge length. Placing this variable control volume randomly in the reconstructed structure and sampling the porosity of the portion of the structure that is enclosed in the corresponding control volume, offered an efficient correlation between the porosity and the volume of the REV.

## 3. Results and Discussion

The successful reproduction of the actual structure is essential to the efficient and realistic description of flow phenomena in the computer-generated samples. A comparison between the geometric properties of the computer-reconstructed structure and those of the experimental data is carried out by visually inspecting and digitally characterizing the reconstructed structures. The suitability of the proposed method, covering a broad range of void fractions between 0.84 and 0.93 and a wide range of pore sizes, was examined for the three foam samples. The ultimate test of the reconstruction efficiency is certainly the comparison of the flow properties with experimental data, as discussed later in this section, given the strong dependence of the flow field and the resulting permeability evaluation on the internal foam structure, especially pore geometry, pore sizes and connectivity.

### 3.1. Domain Reconstruction Results

#### 3.1.1. Structural Features of Reconstructed Foams

**Figure 5:**
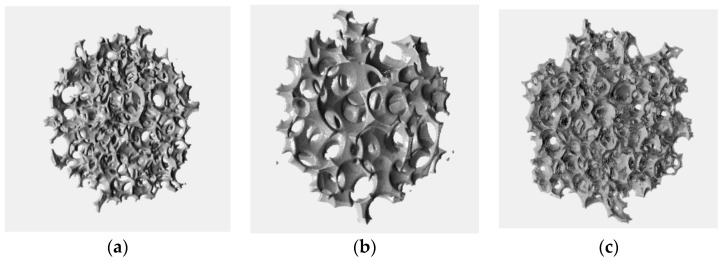
Volume renderings of the reconstructed images. The visualized volume is 250^3^ pixels/voxels which corresponding to cube of edge length of 3.3 mm for (**a**) Gelcast 93 and 1.66 mm for (**b**) Gelcast 87 and (**c**) Gelcast 84.

Qualitatively, the reconstructed images show features similar to the corresponding ones of their reference images, with good connectivity of the void and the solid phases. In a similar manner to their reference images, the reconstructed gelcast foams contain spherical cells with small circular open pore windows interconnecting neighboring cells, as shown in Figure 5a–c, representing the Gelcast93, the Gelcast87 and the Gelcast84 foam, respectively. The spherical cells were a result of the foaming process, as air bubbles were surrounded by Ti_2_AlC particles. The circular pore windows were attributed either to the contact points between bubbles formed in the liquid during the foaming process or due to the fact that the Ti_2_AlC particles were not fully covering the surface of the bubbles during the foaming process. This resulted into uncovered bubble surfaces that were not present during the sintering process [41].

**Figure 6:**
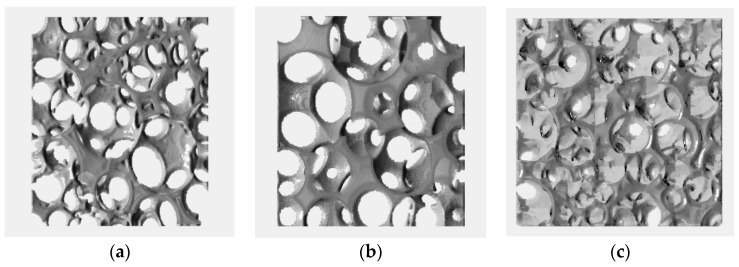
Planar view of the digitally reconstructed foams: (**a**) Gelcast93; (**b**) Gelcast87; (**c**) Gelcast84.

Thin slices of the reconstructed domains reveal that the Gelcast93 was comprised of large spherical cells, with an increased density of pore windows on each cell existed, amounting to an average of 8 pore windows per pore cell, Figure 6a. The Gelcast87 foam, shown in Figure 6b, was also comprised of spherical cells, yet much smaller than in the Gelcast93 foam. Nonetheless, the pore windows density in each cell remained similar. In contrast, the pore cells were much smaller in the Gelcast84 foam, with also much smaller pore window density in each cell, reaching an average of 3.2 pore windows per cell, Figure 6c. The surface cell density is similar to the cell surface density extracted from the SEM image—the Gelcast93, Gelcast87 and Gelcast84 are characterized by a surface cell density of 1.5 cells/mm^2^, 4.0 cells/mm^2^ and 8.5 cells/mm^2^ respectively.

**Figure 7:**
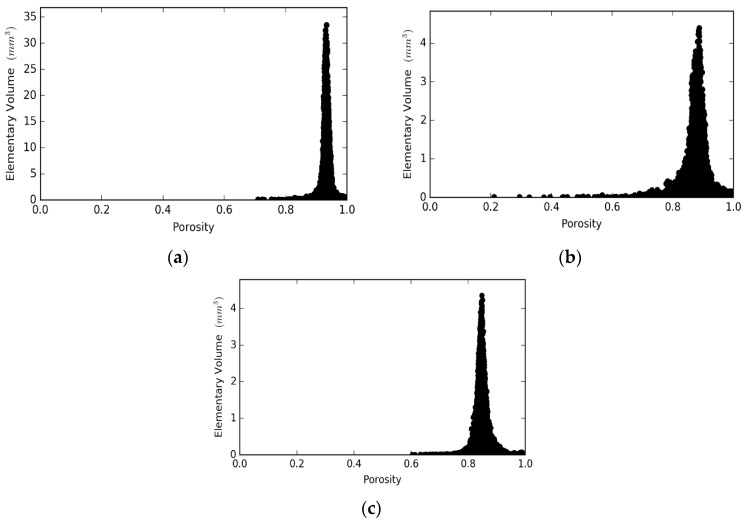
Representative Elementary Volume (REV) analysis for the: (**a**) Gelcast93 foam; (**b**) Gelcast87 foam and (**c**) Gelcast84 foam.

Figure 7 illustrates the REV analysis with respect to the porosity, performed on the generated reconstructed domains of the foams under consideration. The x-axis refers to the porosity of the volume under consideration, while the y-axis gives the volume of the cubic REV. Porosity fluctuated in all systems until it approached a threshold value, where small fluctuations of the porosity over this value were observed. Figure 7b,c show that the REV can have any size between the volume of 3.8 mm^3^ (minimum REV) and 4 mm^3^, the total sample size for the Gelcast84 and Gelcast87 foams. However, in the case of the Gelcast93 foam, a sample size greater than 30 mm^3^ was required, Figure 7a.

#### 3.1.2. Pore Size Distribution of Reconstructed Foams

**Figure 8:**
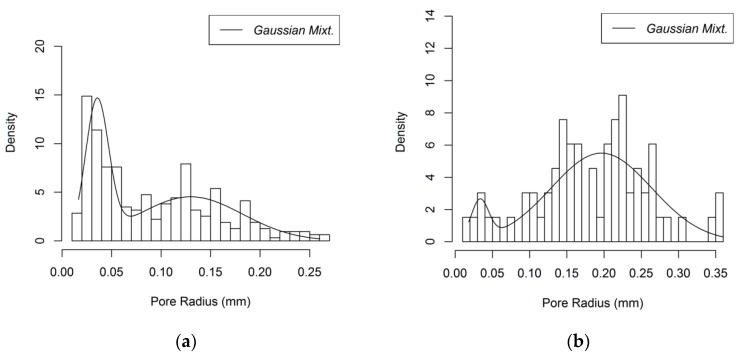

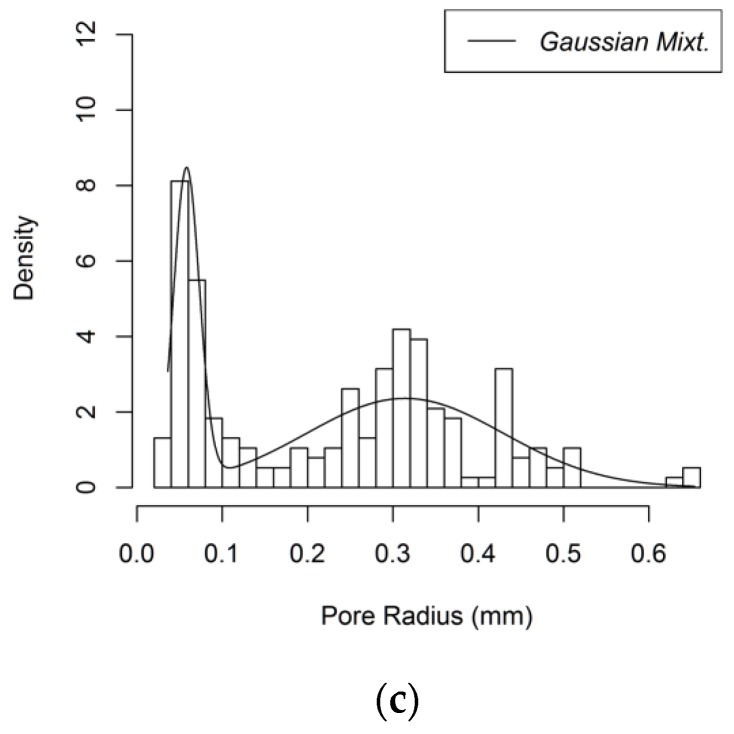
Pore size distribution of (**a**) the Gelcast84 foam; (**b**) the Gelcast87 foam; (**c**) the Gelcast93 foam.

Pores are defined by utilizing the generalized network extraction algorithm developed by Raeini et al. and by assuming a spherical shape to simplify the analysis [42]. The pore size distributions of the Gelcast84, Gelcast87 and Gelcast93 foams are shown in Figure 8a–c, respectively and a statistical description in view of the minimum, the maximum, the median and the mean values of their pore size distributions is given in Table 1.

It is reported in literature that ceramic foams produced by the same gelcasting method are characterized by a bimodal pore size distribution [41], where the large pores are attributed to the foaming process and smaller pores are distributed over the cell walls creating the pore windows, which can be attributable to the contacts among gas bubbles. However, smaller pores can be observed and can be explained by the fact that the powders used for the gelcast generation are not in strict touch with each other; they are distributed over the bubble surface. These powders present a point of contact which tends to be extended during the sintering process, yet it does not fill the entire space among the spheres [32]. The pore size distributions that are obtained from the reconstruction follow a bimodal distribution as shown in Figure 8a–c. The larger part of the macro porosity is attributed to the cells; however, a micro porosity is also present, attributed to the smaller pore windows.

Using the assumption that the macroporosity and the microporosity are independent of each other, the total pore size distribution is regarded as a mixture of the individual pore-size distributions [43]

(24) $$P\left(r_{A}\right)=\lambda_{1}P_{micro}\left(r_{A}\right)+\left(1-\lambda_{1}\right)P_{macro}\left(r_{A}\right),$$

where *r_A_* is a random variable designating the pore radius, λ_1_ is the probability density for a micropore with size *r_A_* and *P_micro_* and *P_macro_* are the micro and macro pore contributions, respectively. The probability densities of the micro and the macro pores, respectively, with a pore radius r*_p_*, follow the Gaussian distribution,

(25) $$P_{micro}\left(r_{p}\right)=\frac{1}{\sqrt{2}\pi s_{d1,P}}e^{\frac{{\left(r_{p}-{µ}_{1,p}\right)}^{2}}{2{\left(s_{d1,p}\right)}^{2}}}\;,\;P_{macro}\left(r_{A}\right)=\frac{1}{\sqrt{2}\pi s_{d2,P}}e^{\frac{{\left(r_{p}-{µ}_{2,p}\right)}^{2}}{2{\left(s_{d2,P}\right)}^{2}}},$$

where *μ*_1,*p*_ and *s*_d1,*p*_ are the mean and standard deviation of the pore radius attributed to the microporosity and *μ*_2,*p*_ and *s*_d2,*p*_ are the mean and standard deviation of the pore radius attributed to the macroporosity. The values for the three foams are given in Table 2.

#### 3.1.3. Characteristic Flow Numbers

For the determination of the dimensional numbers that define the flow regime a strict definition of the characteristic length is required. In this research the hydraulic radius is replaced with the characteristic length of the cross-sectional area of the flow. For one-dimensional flows, the ratio of the cross-sectional area to the wetted perimeter is usually defined as the characteristic length of the flow. By projecting this concept to three dimensions, the characteristic length is defined as the ratio of the total interstitial space to the total interphase surface between the solid and the void phases [44], which is translated to the inverse of the specific surface area of the porous medium. The specific surface area, the characteristic length, the Knudsen, Reynolds and Mach numbers of each foam sample are shown in Table 3. The characteristic length of each foam is similar to its mean pore size shown in Table 1, showing that the utilization of either one as characteristic length would not affect considerably the estimation of the characteristic numbers of the flow. Since the Knudsen number (*Kn*) is smaller than the continuum threshold, the validity of Navier-Stokes equation is assured, whereas, since *Re* is of the order of unity, the laminar flow regime description is valid. Mach numbers (*Ma*) much lower than 0.2 also reveal that compressibility effects are practically not significant for the current flow description.

#### 3.1.4. Domain Meshing

**Figure 9:**
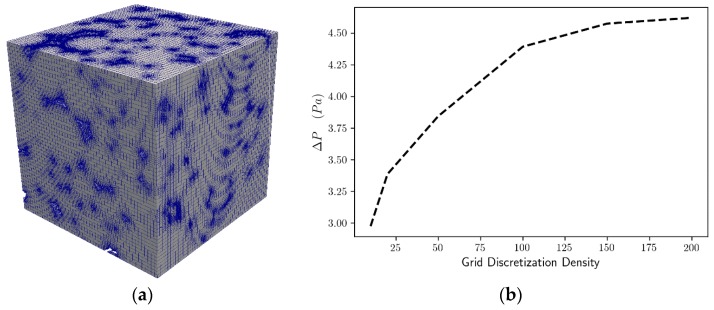
Mesh (**a**) 3D volume rendering of the Gelcast84 foam; (**b**) Grid independence analysis of the mesh for the Gelcast84 foam.

An initial simple hexahedral mesh was created with the blockMesh utility of OpenFOAM. The initial mesh produced by the blockMesh utility was fed in conjunction with the geometries to the snappyHexMesh utility, in order to generate the final hexahedral dominant mesh. The snappyHexMesh utility is an OpenFOAM subprogram that acts simultaneously as a mesh sculptor and a mesh generator [45]. It takes an existing simple mesh like the one generated by the blockMesh and fits the mesh to complex geometries by simultaneously performing mesh refinement and boundary layer addition. The most critical operation was the construction of a high-quality mesh to ensure grid-independent results. To this end, a range of global refinements were tracked throughout the domain to test quantitatively the resulting mesh suitability with the CFD code. The global refinements were performed on the initial hexahedral mesh (using blockMesh) and a range of mesh densities from 25 up to 200 points per dimension was obtained and examined for the mesh independency on the foam with the smaller pore size, namely on the Gelcast84. Local refinement and boundary layers was allowed (using the snappyHexMesh) on each mesh with the same parameters for each resolution. In order to avoid significant numerical errors and extensive use of multiple iterations of non-orthogonal correctors [46], the maximum cell orthogonality in all meshes was set to 65 degrees and the maximum cell skewness was set to 4. The obtained meshes were then tested against their accuracy with respect to the corresponding pressure drop, Figure 9b.

The mesh independence study indicated that a mesh with the domain edge discretized by 150 elements, shown in Figure 9a, was sufficient to capture the essential behavior of the model with suitable accuracy. A comparison of the averaged pressure drop, for a superficial velocity of 0.09 m/s, for the Gelcast84 foam and for different discretization densities is shown in Figure 9b. The results indicate that for 150 elements per edge length of the domain are suitable for these calculations, since larger discretization densities introduce a variation of less than 5%. The most suitable meshes were obtained by the grid independence analysis and their statistics for each foam type are shown in Table 4:

### 3.2. Permeability Estimation

**Figure 10:**
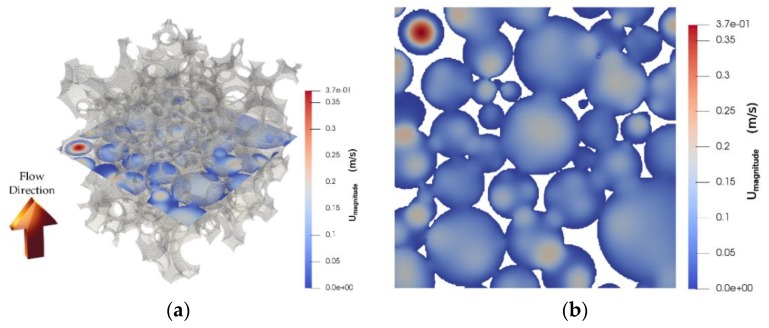
Gelcast93 foam sample. Mid-plane contour plot of the velocity magnitude along the pressure drop (**a**) 3D view in the interior of the foam structure; (**b**) corresponding 2D top view (foam structure removed).

Figure 10a,b illustrates the velocity magnitude distribution of the domain on a through-plane at the middle of the structure, reflecting the flow of air through the spherical pores and the corresponding pore windows. As expected, the bulk of the flow is channeled through high-void space regions, whereas the flow is severely restricted in other areas.

**Figure 11:**
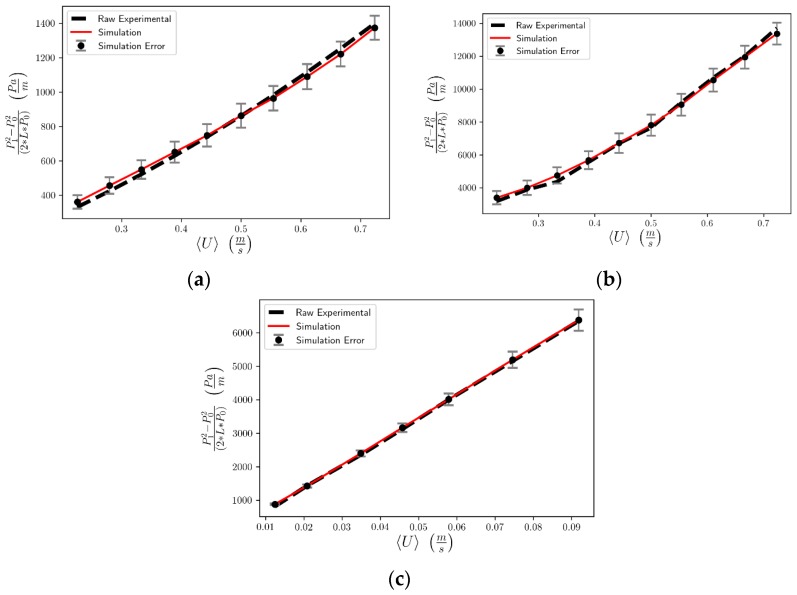
Pressure drop as expressed in References [8,9] vs. superficial velocity for the (**a**) Gelcast84 foam, (**b**) Gelcast87, (**c**) Gelcast93 foams. Comparison of simulation results with experimental data.

In total, four realizations of each foam were produced and the pressure gradient profiles as functions of the mean fluid superficial velocity were obtained and averaged. Figure 11 shows the interdependence of the pressure drop per unit length with the superficial velocity for all three foam samples examined here. A comparison of simulated results with experimental data is made in the figure that reveals a very satisfactory agreement [8,9]. To be more specific, the quality of the fit of the simulated to the experimental data was measured in terms of a normalized root mean square deviation (NRME) and revealed that the NRME value is 0.028 for the Gelcast93 sample, 0.026 for the Gelcast87 and 0.017 for the Gelcast84. Moreover, the variance introduced by the randomness of the foam realizations shows that Gelcast93 exhibits an average deviation of 6%, while Gelcast87 and Gelcast84 exhibits an average deviation of 8% and 4%, respectively. These values are practically very low in view of the complexity of the foam structures.

In this study, the Forchheimer coefficients K_F-D_ and K_F-F_ were obtained by fitting the pressure drop against fluid velocity curves with polynomials of the second order on velocity, with a regression coefficient higher than 0.999 in all cases. The only fitting parameter was the thickness of the pore cells, as discussed above. Values of the simulation and experimental data for the permeability are shown in Table 4 for the sake of direct comparison with each other. The resulting Forchheimer permeability and the estimated inertia coefficient are very close to the experimentally determined values considering that the entire simulation was based on a single SEM image of a small sample of the actual foam.

Moreover, the Darcy permeability and the tortuosity for the corresponding foams were calculated, Table 5. The Stokes flow equation was solved for the determination of the Darcy permeability. The tortuosity was determined by simulating the diffusion of non-sorbing species inside the pore space. More specifically, the diffusion process was simulated by performing discrete lattice walks in the void space [47] and the mean-square displacement of the non-sorbing walkers provided estimation for the diffusive tortuosity. Care was taken in order to ensure that the random walks were conducted under the bulk diffusion regime. This was accomplished by setting the mean free path of the walkers to a sufficiently low value that would correspond to Knudsen number at the continuum limit (0.01 or less).

The Darcy permeability, K_D_, deviated from the experimentally determined Forchheimer permeability, K_F-D,exp_. This observation confirms the conceptual difference between the Darcy and the Forchheimer permeability. The tortuosity values show that the effective diffusivity inside the foams decreases as the foam porosity decreases. Although the Darcy permeability was found to differ by almost two orders of magnitude between the Gelcase93 and Gelcast84, the tortuosity difference was only 32%. This is attributed to the fact that flow is considerably more sensitive to pore size than bulk diffusion.

### 3.3. Pore Wall Thickness

The only fitting parameter of the present algorithm is the wall thickness of the pore cells. However, it was found that the value that was chosen to fit the experimental permeability data was almost identical to the value that could be extracted from the SEM images of all three foam samples, as shown in Table 6. Consequently, the reconstruction method that was developed here could eventually be applied without having to resort to any fitting parameter whatsoever, yet reproducing the experimental data for the flow coefficients of the actual foams shown in Table 7.

### 3.4. Overlap Minimization Aspects

From the technical viewpoint, it must be noted that the SLSQP optimizer was chosen for the minimization of the overlap during the sphere rearrangement process. In theory, SLSQP is appropriate for finding a local minimum of an objective function, however it does not guarantee a solution near a global minimum. Nevertheless, this is not a concern due to the fact that the local minimum obtained by the SLSQP algorithm is still a strong improvement towards the reduction of the overall overlapping. The successive relocation of multiple spheres ultimately reduces the total overlapping volume. Thus, a more complex, global optimization routine to relocate the spheres would not be efficient as it would probably significantly increase the calculation time without an essential improvement in the final result.

### 3.5. Limitations and Future Work

The proposed reconstruction algorithm focuses on the reconstruction of isotropic ceramic foams produced with the gelcast method; however, for a plethora of foamy materials, such as multiscale polylactic acid structures (with largely varied secondary porosity and pore radii), pore shape distribution and pore size vary greatly. Building a precise 3D digital reconstruction using a single 2D slice is rather difficult. This is attributed to the inability to ensure the representative adequacy of the 2D images at hand.

Moreover, in the proposed reconstruction algorithm the shape of the pores, as well as the shape of the pore windows, are assumed to be spherical. This assumption seems to be valid in the majority of the foams produced with the gelcast method, since the pore shapes are nearly spherical. If pore cells strongly deviate from the spherical shape (e.g., foamy layers produced with the phase inversion method), the proposed approach can be extended to an arbitrary pore shape, provided that there is an analytical expression of the overlapping volume between the pore objects.

## 4. Conclusions

A 3D reconstruction method was developed in this work, aiming at the stochastic digital representation of the internal structure of ceramic foams that are produced by the gelcast method using a single SEM foam image. The only fitting parameter that was employed was the thickness of the pore walls, while all other geometrical and topological factors were directly matched to the experimentally extracted ones from image processing. The pore wall thickness can be treated as the single fitting parameter in the entire reconstruction process, although it is shown that images of sufficient resolution could eliminate the need even for that. It was also shown that the pore wall thickness was very close to the value that could be extracted from the SEM images of all three types of foams examined here.

The novel hybrid algorithm developed here combines a variation of Lubachevsky-type and Random Close Packing of Hard Spheres algorithms to obtain near-optimum solutions to the pore-sphere packing problem. The proposed method ensures that the pore cell size distribution matches the one extracted from the imaging data, a factor missing from previous tessellation-related works. In addition, the proposed method approaches the curvature of inherently spherical foam cells using spheres rather than deformed polyhedra, as implemented in previous reconstruction methods.

The bimodal porosity distribution of the foams, in view of the macrovoids of the cells and the smaller porosity of the interconnecting pore windows, is captured with the proposed algorithm for all three types of foams examined here, in accord with experimental observations related to their internal structure description. Moreover, a key advantage of the proposed reconstruction algorithm is its ability to reconstruct realistic foam structures with pairs of pore cavities interconnected via multiple pore windows, a feature that cannot be reproduced with typical tessellation processes.

Numerical simulations are performed directly on the 3D reconstructed foams to determine their gas permeability. The model predictions are compared with experimental gas permeability data that were obtained for the physical samples, showing a satisfactory agreement, as they produced quantitatively similar pressure drops with experiments under variable superficial velocity, with a residual deviation, quantified by the normalized root mean square deviation (NRME), in the very low range of 1.7–2.8%. Moreover, the successful repeatability of the reconstruction method is shown in the low value of the deviation introduced by the randomness of the foam realizations, which shows that gelcast foams studied here exhibit a low deviation in the range of 4–8%.

The difference between the first-order Forchheimer term and the Darcy permeability was confirmed by direct calculation of their values for the same computational domains. Despite the complexity of the structure of these foams, it was found that the 3D reconstruction of pore cells and pore windows connecting the cells was successful judging from the calculation of the flow properties of the samples.

Overall, the proposed reconstruction process can assist in a rapid characterization of the foams produced by the gelcast method, avoiding most of the time-consuming experimental characterization processes. With information extracted solely from a few SEM images, such as the pore size and the window size distribution and the pore wall thickness, the user can obtain an accurate 3D digital representation of the foam at hand. The 3D reconstructed volumes can be further utilized in order to obtain valuable structural information, such as the specific surface area, as well as their corresponding effective resistance to mass transport and flow.

## Figures and Tables

**Table 1:** Statistics of the pore size distribution of the three different foams.

Foam	Min	Max	Median	Mean
	(mm)	(mm)	(mm)	(mm)
Gelcast84	0.017	0.26	0.075	0.091
Gelcast87	0.004	0.36	0.194	0.182
Gelcast93	0.036	0.65	0.254	0.233

**Table 2:** Mean and standard deviation of the microporosity and macroporosity of the foams examined and the probability density for the micropores.

Foam	*μ*_1,*p*_	*s*_d1,*p*_	*μ*_2,*p*_	*s*_d2,*p*_	*λ_1_*
	(mm)	(mm)	(mm)	(mm)	
Gelcast84	0.049	0.027	0.120	0.049	0.94
Gelcast87	0.096	0.039	0.184	0.053	0.87
Gelcast93	0.058	0.015	0.310	0.110	0.89

**Table 3:** Specific surface area and resulting characteristic numbers of the foam simulations.

Foam	Specific Surface Area (SSA) (m^2^/m^3^)	Characteristic Length (mm)	*Kn*	*Re*	*Ma* (×10^−4^)
			(×10^−4^)	Min-Max	Min-Max
Gelcast93	3090	0.323	1.75	1.07–7.52	2.9–3.0
Gelcast87	4480	0.223	2.5	0.74–5.18	2.9–3.0
Gelcast84	11272	0.088	21.01	0.02–0.22	0.29–0.3

**Table 4:** Summary of the cell type distribution of the three different foams.

Foam	Total Cell Counts	Hexahedra	Prisms	Tet Wedges	Polyhedra
Gelcast93	4603068	3395049	150086	19190	1038743
Gelcast87	5576328	4106789	212812	5656	1251071
Gelcast84	6232131	4532497	293359	5816	1400459

**Table 5:** Estimated and Experimental Forchheimer Coefficients.

Foam	*K_F_—D,_exp_*	*K_F_—D,_sim_*	*K_F_—F,_exp_*	*K_F_—F,_sim_*
	Experimental Forch.-Darcy [8,9]	Estimated Forch.-Darcy	Experim.	Estimated
			Inertia Coef. [8,9]	Inertia Coef.
Gelcast93	1.79 × 10^−8^ m^2^	2.99 × 10^−8^ m^2^	1.75 × 10^−3^ m^−1^	1.62 × 10^−3^ m^−1^
Gelcast87	2.05 × 10^−9^ m^2^	2.16 × 10^−9^ m^2^	7.38 × 10^−5^ m^−1^	7.13 × 10^−5^ m^−1^
Gelcast84	9.34 × 10^−10^ m^2^	9.34 × 10^−10^ m^2^	3.46 × 10^−5^ m^−1^	3.54 × 10^−5^ m^−1^

**Table 6:** Wall thickness of foam cells. Comparison of fitted value and actual one.

Foam	Wall Thickness Simulated	Wall Thickness Experimental [8,9]
Gelcast93	9.87 μm	9.45 ± 0.6 μm
Gelcast87	10.11 μm	11.53 ± 1μm
Gelcast84	20 μm	22± 0.4 μm

**Table 7:** Numerically estimated tortuosity and Darcy permeability.

Foam	*τ*, Tortuosity	*K_D_*, Darcy Permeability
Gelcast93	1.126	1.05 × 10^−8^ m^2^
Gelcast87	1.210	3.70 × 10^−9^ m^2^
Gelcast84	1.495	7.02 × 10^−10^ m^2^
